# A Validated Stability-Indicating HPLC Method for Routine Analysis of an Injectable Lincomycin and Spectinomycin Formulation

**DOI:** 10.3797/scipharm.1207-13

**Published:** 2012-09-10

**Authors:** Murad N. Abualhasan, Nidal Batrawi, Oliver B. Sutcliffe, Abdel Naser Zaid

**Affiliations:** 1 Department of Pharmacy, An-Najah National University, Nablus, Palestine.; 2 Advanced Veterinary Company, Ramallah, Palestine.; 3 Division of Chemistry & Environmental Science, School of Science and the Environment, Manchester Metropolitan University, Manchester, United Kingdom.

**Keywords:** Stability-Indicating, RP-HPLC, Lincomycin, Spectinomycin

## Abstract

Lincomycin and spectinomycin combination therapy is widely used in veterinary medicine for the treatment of gastrointestinal and respiratory infections caused by lincomycin- and spectinomycin-sensitive microorganisms. A simple, reverse phase HPLC method for the analysis of samples of an injectable lincomycin and spectinomycin preparation containing a mixture of inactive excipients has been developed. The HPLC was carried out using the RP-C_18_ (250 mm × 4.0 mm, 5 μm) column, with the gradient mobile phase consisting of an acetonitrile and phosphate buffer at pH 6; the flow rate of 1 mL/min and ultraviolet detection at 220 nm. This method was validated in accordance with both FDA and ICH guidelines and showed good linearity, accuracy, precision, selectivity, and system suitability results within the acceptance criteria. A stability-indicating study was also carried out and indicated that this method can also be used for purity and degradation evaluation of these formulations.

## Introduction

A formulation containing a combination of both lincomycin (a lincosamide-antibiotic derivative, produced by the microorganism *Streptomyces lincolnensis*) and spectinomycin (aminoglycoside-like antibiotic produced by *Streptomyces spectabilis*) is widely used in veterinary medicine for the treatment of gastrointestinal and respiratory infections caused by lincomycin and spectinomycin-sensitive micro-organisms, like *Campylobacter*, *E. coli*, *Mycoplasma*, *Salmonella*, *Staphylococcus*, *Streptococcus*, and *Treponema sp.* in a wide variety of animals including: calves, cats, dogs, goats, poultry, sheep, and turkeys [1–4].

There are a number of analytical methods for the quantification of both spectinomycin [5–7] and lincomycine [8, 9] as single agents reported in the literature. In addition to these methods, the European Pharmacopoeial monograph for spectinomycin includes an LC-EC method to detect both the antibiotic and its production impurities [10], however a simple, routine QC/QA chromatographic assay for the analysis/quantification of both agents within a combined formulation remains unexplored. The simultaneous determination and analysis of both antibiotic agents in a single formulation is complicated due to the difference in the chromatographic behaviour of these two active ingredients. The only reported methods for the determination of the two agents in a formulation employs reverse phase liquid chromatography (RP-LC) coupled to a specialised Pulsed Amperometric Detector (PAD) utilising a gold electrode [11] or highly specialised bioanalytical methods [12–15], both of which are unsuitable for routine QA/QC applications.

This study reports the development and validation of a simple reverse phase chromatographic method using UV detection for the routine analysis and quality control of the combined spectinomycin and lincomycin injectable formulation which contains the active ingredients: lincomycin hydrochloride (LH, 50 mg/mL) and spectinomycin sulphate (SS, 100 mg/mL), and also a mixture of inactive excipients manufactured by the Advanced Veterinary Company in Ramallah-Palestine. The advantage of this proposed protocol over previously reported methods is its simplicity and broad applicability using a standard HPLC system and UV detection. The active ingredient and its related compounds showed good absorption at the lower wavelength of 220 nm. The mobile constituents of pH 6 probably improved the absorptive power of these active ingredients and hence, increase its limit of detection. The method can be used for both routine quality control/assurance of both of the active ingredients in a short, single run and assessment of drug–excipient stability and compatibility within the formulation. The method, detailed herein, has been fully validated in accordance with the requirements of the FDA and the ICH guidelines [16].

## Experimental

### Instrumentation

Chromatographic analysis was carried out using a Dionex-Ultimate 3000 HPLC system equipped with an LPG-3400SD pump, WPS-3000SL autosampler, TCC-3000 column oven, and DAD-3000 UV–VIS diode array detector. Chromeleon Datasystem Software (Version 6.80, DU10A Build 2826, 171948) was used for data acquisition and analysis.

### Material

Lincomycin hydrochloride (LH) and spectinomycin sulphate (SS) were purchased from Quingdao Dacon Trading Co., China. The USP reference standards of the two agents were used as working reference standards. The injection dosage form lincomycin hydrochloride (50 mg/mL) and spectinomycin sulphate (100 mg/mL) was manufactured by the Advanced Veterinary Company in Ramallah-Palestine. Acetonitrile (HPLC grade), phosphoric acid, hexanesulfonic acid sodium salt, ammonium hydroxide, hydrochloric acid (0.5 N), sodium hydroxide (0.2 N), and 3% hydrogen peroxide were obtained from commercial suppliers. HPLC grade water was obtained through double distillation via Aquatron distillation equipment (Model A 4000D).

### Chromatographic conditions

The phosphate buffer was prepared by dissolving phosphoric acid (13.5 g) and hexanesulfonic acid sodium salt (600 mg) in 800 mL of distilled water. The pH of the solution was adjusted to 6.0 ± 0.1 with aqueous ammonium hydroxide and then made up to the volume (1000 mL) with distilled water. The initial mobile phase buffer-acetonitrile [89:11 % v/v] was employed for the preparation of the standard solutions and samples for analysis. The gradient programme and HPLC conditions employed in the study is detailed in Tables 1 and 2.

### Preparation of stock solutions

Lincomycin hydrochloride (283 mg) and spectinomycin sulphate (500 mg) were weighed accurately into a 50 mL volumetric flask and dissolved in the buffer-acetontrile [89:11 % v/v] (standard stock solution). The samples were prepared by diluting (1:10) the injection formulation with the buffer-acetontrile [89:11 % v/v] to yield solutions with concentrations equivalent to the standard stock solution.

## Method validation and degradation studies

The method was validated in accordance with the FDA and ICH guidelines using the following parameters: specificity, linearity, range, accuracy, precision, and ruggedness/robustness. Forced degradation studies were performed to evaluate the stability-indicating ability and specificity of the method. Intentional degradation of the formulation was carried out under acidic (0.1 N HCl), basic (0.2 N NaOH), oxidising (3% H_2_O_2_), photolytic, and thermal conditions (Table 3). The stressed samples were analysed periodically and the related peaks were monitored in terms of their retention times, peak interference, and resolution factor.

Linearity and the range of the method was evaluated using the following concentration range (based upon the original formulation): 60% (3 mg/mL LH; 6 mg/mL, SS), 80% (4 mg/mL LH; 8 mg/mL, SS), 100% (5 mg/mL LH; 10 mg/mL, SS), 120% (6 mg/mL LH; 12 mg/mL, SS), and 140% (7 mg/mL LH; 14 mg/mL, SS). Ten separate injections were diluted (1:10) and analysed under the same conditions.

Accuracy and precision were established by analysis of three concentrations near the test concentration (80%, 100%, and 120%) (three replicates of each concentration). The percentage recovery and %RSD were calculated for each of the replicate samples.

The ruggedness/robustness of the method was determined by performing the same analysis using minor modifications of the method, for example: a different mobile phase pH, detection wavelength, flow rate, elapsed assay time, and analyst. The applied ruggedness parameters are illustrated in Table 4.

## Result and discussion

### Linearity and Range

**Fig. 1.**
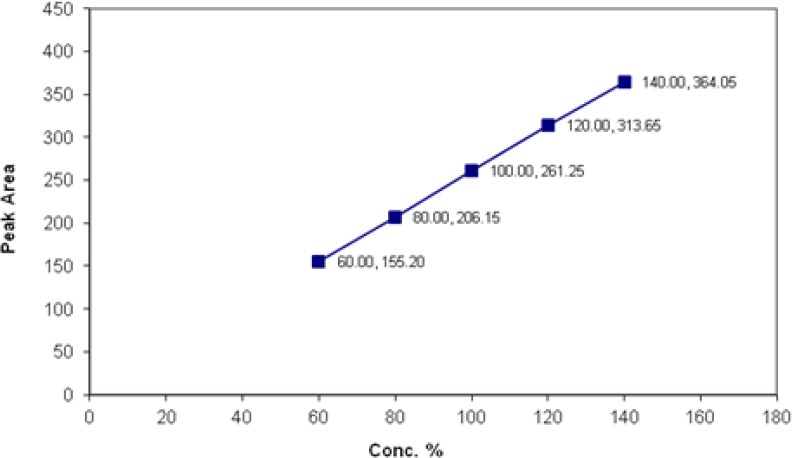
Linearity and range of spectinomycin sulphate

**Fig. 2.**
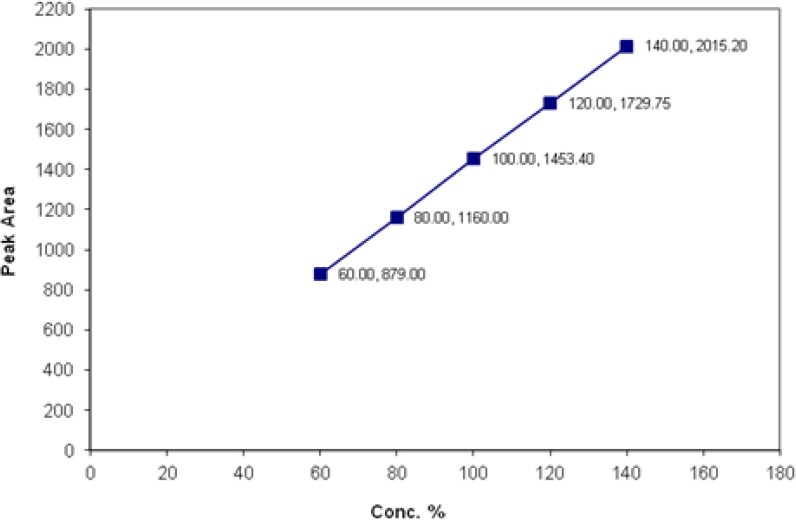
Linearity and range of lincomycin hydrochloride

The linearity of the method was demonstrated over the concentration range (60% to 140%) for both spectinomycin and lincomycin, demonstrating its suitability for analysis as shown in Figures 1 and 2. The goodness-of-fit (R^2^) was found to be 0.9999 indicating a linear relationship between the concentration of the analyte and the observed peak area. The slopes of the regression line (b coefficient) for spectinomycin and lincomycin were 2.6 and 14.2, respectively, and both of them were statistically significant with a P value <0.01.

### Specificity and stability-indicating study

Stress testing of the spectinomycin and lincomycin injection formulation was undertaken to determine the stability of the molecules themselves, identify the likely degradation products, and to validate the specificity of the analytical procedure. The stability-indicating studies were performed under a variety of stress conditions (see Method validation and degradation studies). The results of the specificity studies (Table 5) indicated no interference from the excipients, impurities, and degraded products under the various stress conditions.

**Fig. 3.**
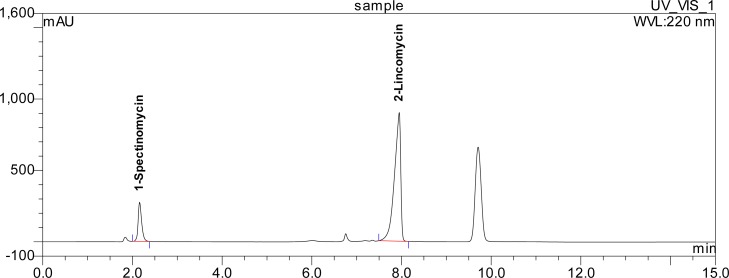
Chromatogram of well separated peaks of the active ingredient, degradative peaks, and the injections formulation. Note: The small peaks prior to the active ingredients are degradative peaks and the late eluting peak is the preservative benzyl alcohol.

The active pharmaceutical ingredients (APIs) under acidic conditions demonstrated a significant reduction in the active ingredient peak areas with the concomitant development of new peaks for the degradation products (A) (ca. 6.5%) and (B) (ca. 8%), with respect to the original peak, for spectinomycin and lincomycin respectively. The sample degraded in alkaline solution as demonstrated by the reduction of the spectinomycin peak area and a peak corresponding to degradation product (C), developing over the course of seven days. Spectinomycin showed degradation under the thermal stress conditions. The formulation demonstrated relatively good stability under oxidative or photolytic (UV light) conditions. In all cases, the peaks of the degradation products were completely resolved from the peaks for the active ingredients in the formulation. The purity of all the peaks was tested and the results showed that none of the tested peaks had a purity of less than 99% (Figure 3).

### Accuracy and Precision

The method demonstrated excellent accuracy within the desired concentration range. The %RSD was calculated for the percentage recovery of each test solution for both active ingredients. All the results are within acceptable limits (100±2%) and the data is summarised in Tables 6 and 7.

### Ruggedness/Robustness

The ruggedness and robustness of the method were examined using the minor modifications detailed in Table 4. The data obtained indicates that minor modifications to the experimental parameters do not affect the assay and its ability to accurately and precisely detect/quantify the active ingredients.

### Detection and quantification limit (LOD &LOQ)

The detection limit or LOD is the lowest amount of analyte in a sample that can be detected. It may be expressed as a concentration that gives a signal-to-noise ratio of approximately 3:1. While the limit of quantification or LOQ is the lowest amount of analyte in a sample that can be determined with acceptable precision and accuracy with a signal-to-noise ratio of approximately 10:1 can be taken as LOQ. Our method showed an (LOD) of 0.042 & 0.004 mg/mL for spectinomycin and lincomycin respectively. The LOQ was 0.14 & 0.013 mg/mL for spectinomycin and lincomycin respectively.

### System Suitability

System suitability tests are used to verify that a system is performing adequately to ensure confidence in the analytical method and the results obtained. The developed method shows that all of the standard system suitability parameters are within acceptable limits (Table 8). Column efficiency (N) was determined to be 3450 and 16500 theoretical plates for spectinomycin and lincomycin respectively. The peak symmetry/tailing factors were determined to be 1.1 and 0.67 for spectinomycin and lincomycin respectively. The resolution between the peaks for the two active ingredients were determined to be 2.4 (for spectinomycin) and 6.7 (for lincomycin) respectively.

## Conclusion

A new, robust, and suitable HPLC assay utilising UV detection has been developed for the analysis of an injectable lincomycin-spectinomycin combination. This method has several advantages over other known methods for the analysis of these active ingredients; it is economical and can be used for the rapid assay of the two ingredients simultaneously. The developed method was validated in accordance with both FDA and ICH guidelines and shows excellent linearity, accuracy, precision, selectivity, and system suitability within the acceptance criteria. The method has been applied in stability-indicating studies and indicates that this method is suitable for both purity and degradation evaluation applications.

## Authors’ Statement

### Competing Interests

The authors declare no conflict of interest.

## Figures and Tables

**Tab. 1.** Gradient elution programme

**Duration (min.)**	**Buffer**		**acetonitrile**	
	**Start**	**End**	**Start**	**End**
0–2	89	89	11	11
2–3	89	78	11	22
3–8	78	78	22	22
8–9	78	89	22	11
9–15	89	89	11	11

**Tab. 2.** HPLC chromatographic conditions

**Chromatographic conditions**	
Flow rate	1.0 mL/min
Wavelength (λ)	220 nm
Stationary phase	RP18e, 5 μm, 250 × 4 mm
Column Temperature	35°C
Injection volume	20 μL
Run time	15 minutes.

**Tab. 3.** Stress conditions

**Stress type**	**Conditions**	**Time**
Acidic hydrolysis	1 mg/mL in 0.1N (up to 1N), HCl at 65 °C	1–7 days
Basic hydrolysis	1 mg/mL in 0.1N (up to 1N), NaOH at 65 °C	1–7 days
Oxidative	0.3% (up to 3%) H_2_O_2_; at RT; protected from light	Few hours to 7 days
Thermal	70°C (RH of 40%)	Up to 3 weeks
Photolytic degradation	UV light (254 nm at room temperature)	Few hours to 3 days

**Tab. 4.** The applied ruggedness/robustness conditions

**Robustness parameter**	**Condition checked**
pH values of the mobile phase	pH of the mobile 5.9, 6.0 & 6.1
Detection wavelength	WL of 218, 220 and 222 nm
Flow rate of the mobile phase	Flow rate of 0.8, 1.0 and 1.2 mL/min
Elapsed assay times	The same analyst analysed the same trial in two different days
Analysts	Two analysts analysed the same trial in the same day

**Tab. 5.** The results of specificity and stability-indicating studies.

**Stress type**	**Detectable change**
Acidic hydrolysis	Both major peaks area loss and degradation products (A) and (B).
Basic hydrolysis	Degradation product (C).
Oxidative	No change
Thermal	Loss of spectinomycin peak area
Photolytic degradation	No change

**Tab. 6.** Accuracy and precision results of spectinomycin

**Sample**	**Sample Peak Area**			**Standard Peak Area**			**Assay (%)**

**No.**	**inj # 1**	**inj # 2**	**average**	**inj # 1**	**inj # 2**	**average**	
**80%**							

1	250.00	251.00	250.50	253.00	251.00	252.00	99.40
2	250.00	251.00	250.50				99.40
3	250.00	250.00	250.00				99.21

**100%**							

1	311.00	311.00	311.00	312.00	312.00	312.00	99.68
2	315.00	315.00	315.00				100.96
3	314.00	313.00	313.50				100.48

**120%**							

1	376.00	376.00	376.00	375.00	375.00	375.00	100.27
2	376.00	376.00	376.00				100.27
3	374.00	374.00	374.00				99.73
**Mean**							99.93
**SD**							0.59
**RSD**							0.59

**Tab. 7.** Accuracy and precision results of Lincomycin

**Sample**	**Sample Peak Area**			**Standard Peak Area**			**Assay (%)**

**No.**	**inj # 1**	**inj # 2**	**average**	**inj # 1**	**inj # 2**	**average**	
**80%**							

1	1204.00	1204.00	1204.00	1195.00	1193.00	1194.00	100.84
2	1201.00	1201.00	1201.00				100.59
3	1201.00	1201.00	1201.00				100.59

**100%**							

1	1485.00	1485.00	1485.00	1476.00	1477.00	1476.50	100.58
2	1497.00	1497.00	1497.00				101.39
3	1494.00	1493.00	1493.50				101.15

**120%**							

1	1770.00	1770.00	1770.00	1754.00	1754.00	1754.00	100.91
2	1772.00	1771.00	1771.50				101.00
3	1783.00	1783.00	1783.00				101.65
**Mean**							100.97
**SD**							0.38
**RSD**							0.37

**Tab. 8.** System suitability

	**Spectinomycin**	**Lincomycin**	**Acceptance Criteria**
Resolution (R)	2.4	6.7	> 1.5
Tailing (T)	1.16	0.65	≤2.0
Theoretical plates (N)	3450	16500	≥1000 plates
